# Evolution of contrast agents for ultrasound imaging and ultrasound-mediated drug delivery

**DOI:** 10.3389/fphar.2015.00197

**Published:** 2015-09-15

**Authors:** Vera Paefgen, Dennis Doleschel, Fabian Kiessling

**Affiliations:** Institute for Experimental Molecular Imaging, RWTH Aachen University Hospital, AachenGermany

**Keywords:** ultrasound, contrast agent, microbubbles, nanobubbles, molecular imaging, drug delivery, theranostics

## Abstract

Ultrasound (US) is one of the most frequently used diagnostic methods. It is a non-invasive, comparably inexpensive imaging method with a broad spectrum of applications, which can be increased even more by using bubbles as contrast agents (CAs). There are various different types of bubbles: filled with different gases, composed of soft- or hard-shell materials, and ranging in size from nano- to micrometers. These intravascular CAs enable functional analyses, e.g., to acquire organ perfusion in real-time. Molecular analyses are achieved by coupling specific ligands to the bubbles’ shell, which bind to marker molecules in the area of interest. Bubbles can also be loaded with or attached to drugs, peptides or genes and can be destroyed by US pulses to locally release the entrapped agent. Recent studies show that US CAs are also valuable tools in hyperthermia-induced ablation therapy of tumors, or can increase cellular uptake of locally released drugs by enhancing membrane permeability. This review summarizes important steps in the development of US CAs and introduces the current clinical applications of contrast-enhanced US. Additionally, an overview of the recent developments in US probe design for functional and molecular diagnosis as well as for drug delivery is given.

## Introduction

Ultrasound (US) imaging enables cheap, non-invasive imaging in real-time with a high soft tissue contrast and without exposing the patient to radiation. This, together with its broad field of applications, explains the extensive use of clinical US imaging. Applications range from first-look examinations in abdomen or extremities to cardiac applications and endosonography, e.g., in the female genital tract. US regularly supplements x-ray mammography and it is used for the assessment of tissue vascularization and of vessel occlusion using Doppler. In many cases, additional CA are not needed to find the right diagnosis. For poorly vascularized tumors or regions with many small vessels with slow blood flow Doppler US is not sufficient. Hence CEUS is an option and was shown to improve cancer detection and tumor characterization, decreasing the number of biopsies, or during surgery in brain cancer patients (Kitano et al., 2012; Uemura et al., 2013; Prada et al., 2014).

Application of CEUS started in the late 1960s after finding that the injection of agitated saline caused a detectable signal change during US examination (Gramiak and Shah, 1968). Contrast enhancement was caused by the compressible gas core of saline bubbles, enabling the bubble to backscatter the applied US wave. Those first saline bubbles were unstable due to the high surface tension. By injection of autologous blood at adequately rapid rates, the formation of more stable bubbles was described (Kremkau et al., 1970), nonetheless those bubbles still lacked sufficient lifetime and a defined size. It took more than 20 years to develop the first stable, commercially available and FDA-approved USCA (Feinstein et al., 1990), Albunex^®^, an albumin-coated and air-filled microsphere.

Since then, stability and biocompatibility of USCA have been continuously improved and bubbles have been modified to specifically target certain surface molecules expressed in pathological alterations. Apart from their support for imaging and diagnostics, micro- and NBs are object of increased interest for therapeutic applications. Recent studies used the disrupting effect of MB-enhanced US on the BBB in combination with transplantation of mesenchymal stem cells for treatment of brain ischemia, or used MBs as carriers of drugs, siRNA and mRNA (Dewitte et al., 2014; Gong et al., 2014). This broad field of different uses makes USCA attractive for research and beneficial for patients. Currently three different MB-based CA are clinically approved in the United States/North America and Europe, and a fourth is clinically used in Japan and South Korea, but the variety among the investigative CA is much broader and frequently produces new, promising progenies. Since examinations with those approved CA are common in the clinics, guidelines for CEUS imaging of the liver exist to guarantee proper and comparable examinations and an improvement for the patients’ diagnosis and therapy (Claudon et al., 2013).

## Diagnostics

Due to their broad applicability and low risks, many different types of USCA have been developed. To get started, an overview of the different possibilities to use bubbles of varying size, shell material, or gas cores will be presented, as well as their properties, applications, and advantages.

### Microbubbles

The majority of USCA in use are MBs. As their name suggests, their diameters range between 1 and 10 μm. This size normally limits the application of MB to the intravascular system to assess functional parameters like vascularity, perfusion, blood flow velocity, angiogenicity, or to characterize vasculature molecularly by using targeted MB (Yuan and Rychak, 2013). Extravasation of MB to surrounding tissue is inhibited, preventing unspecific accumulation in the interstitial space and unwanted background signals. Micron-sized bubbles were found to cause proper backscattering of applied US pulses, not only with linear oscillations, but also with non-linear ones, which are not strongly present in most tissues. Thus, MB can be detected with high sensitivity and a good contrast.

In this review we will introduce the main different shell materials, normally divided in soft- and hard-shell bubbles, will be introduced. Even though the included gas is responsible for the majority of the bubbles’ acoustic properties, the shell adds a mechanical stiffness and reduces the compressibility of the gas. Therefore, the shell material provides multiple possibilities to tailor the MB to their specific application by changing visco-elastic properties (Hoff et al., 1996; Kiessling et al., 2014).

Nonetheless, the choice of gas is a factor that has to be considered. First experiments used air, but still suffered from poor stability and very short circulation times due to the high solubility of air in water. The same difficulties occurred in tests using a nitrogen filling, though a less gas-permeable coating slightly improved lifetime (Unger et al., 1994; Schutt et al., 2003). It was found that PFC were a good choice with their low solubility in water/blood and their good compatibility. With introduction of PFC it became possible to produce MB of a defined size with a lifetime of several minutes, long enough for diagnostic examinations *in vivo*. To rule out possible changes in bubble-size by air diffusing along the concentration gradient between blood and bubble, MB can be produced with a defined mixture of PFC and air, so that Laplace and arterial pressure are in equilibrium (Schutt et al., 1996). Another useful side effect of using PFC is the possible application of those USCA for MRI, since fluorine (19F) is NMR-detectable, even with a normal, slightly adjusted proton setup (Siemens TIM Trio 3T MRI scanner, transmit/receive 19F/1H dual-tune volume RF coil, a pre-amplifier), and does not cause background signals in patients (Rapoport et al., 2011). However, the amount of fluorine to detect is extremely small and requires a highly sensitive setup.

#### Hard-Shell Microbubbles

The group of HS-MBs mainly consists of gas bubbles with a coating of lower visco-elastical properties such as polymers or denatured proteins, as well as porous silica materials encapsulating gas. Generally, HS-MB show an increased circulation time *in vivo* and are the preferred type of CA for higher-intensity US applications where they provide a higher echogenicity than SS-MBs which might rupture.

##### Polymer-shelled microbubbles

The first polymers used for US applications were naturally occurring air-filled polymers. Gelatin was among the first biopolymers to be tested, but the production of adequately small MB turned out to be difficult and their circulation time was short (Carroll et al., 1980). Wheatley et al. (1990) pointed out the lack of appropriate CA for US diagnostics 20 years after the discovery of gas bubbles as a suitable system. They developed a setup for alginate-air MB, but again struggled with the needed maximal size of approximately 10 μm (Wheatley et al., 1990). Other approaches used agarose gel as shell material (D’Arrigo, 1981) with similar complications. For all natural polymers an increased risk of material contaminations was found, in addition to decreased reproducibility of size-defined bubbles and adequate *in vivo* stability to enable clinical examinations (Cohen et al., 1996; Schutt et al., 2003). Until today, there are no clinically approved CA derived from those natural polymers, though a few groups still work with those materials due to their good compatibility (Huang et al., 2013). By switching to synthetic polymers, the risk of contamination could be decreased and many of them have proven their biocompatibility in other applications (Kelly et al., 2003). Modern polymer-shelled MB are normally made of synthetic polymers with a PFC/air-filling.

Cyanoacrylate polymers were first used as a shell material by Fritzsch et al. (1994). Under the name SHU 563 A, later on Sonovist^®^, (Schering AG, Berlin), those air-filled MB were shown to last more than 10 min *in vivo*, both in animals and in patients, to have a good biocompatibility and to be taken up by the reticuloendothelial system effectively. These properties made SHU 563 A an interesting tool for liver and spleen US examination (Bauer et al., 1999; Forsberg et al., 1999). Apart from Sonovist^®^, other cyanoacrylates are investigated for US applications as well. PBCA is a well-known biocompatible polymer and, as a gas-filled MB, tested for various diagnostic or therapeutic US-supporting applications. Synthesis of PBCA-MB includes intensive stirring during polymerization in presence of a detergent like Triton X-100 and hydrochloric acid (pH = 2). Olbrich et al. (2006) compared one- and two-step synthesis protocols and stirring intensity to vary size, shell thickness and the resulting properties of MB under acoustic pressure, as well as the MB survival time in plasma and serum. Factors that might interfere with MB stability were high injection rates and small needle diameters during MB injection, but when handled and stored correctly, PBCA-MB remained stable for multiple weeks (Fokong et al., 2011). Besides using PBCA-MB for drug delivery in therapeutic approaches, they can also be labeled with fluorophores or iron nanoparticles and thus be a useful tool for multimodal imaging with US and 2-photon-/fluorescence microscopy or MRI (Koczera et al., 2012; Lammers et al., 2015).

Poly(D,L-lactide-co-glycolide) is another commonly used material for MB synthesis. Here, bubbles are produced from a double emulsion of water, oil, and water, followed by evaporation of solvents. Compared to PLLA, PLGA has a faster degradation rate (Cui et al., 2005a). By varying factors like the molecular weight or adding capping structures to the polymers’ ends, US scattering properties could be adjusted (Eisenbrey et al., 2008). Among the first animal experiments for PLGA-MB, myocardial contrast echocardiography was successfully done in dogs (Cui et al., 2005b). More recent approaches make use of PLGA-MB as delivery vectors, often in tumor treatment. Niu et al. (2012, 2013) used PLGA-MB to support identification of lymph nodes near tumor sides during surgery by delivering Sudan black, as well as the chemotherapeutic drug doxorubicin and iron particles, to make use of both US and MRI evaluation. PLGA-MB have been used as CA with sonosensitizer properties for tumor treatment as well, when coupled to hematoporphyrin that gets activated by US and is supposed to induce tumor necrosis (Zheng et al., 2012). Nonetheless there are currently no FDA-approved PLGA-MB for clinical applications.

Poly(vinyl-alcohol) is characterized by a good biocompatibility and its hydroxylic moiety which allows multiple chemical modifications (Cavalieri et al., 2005). Around 10 years ago several groups started working on PVA-MB. Similar to PBCA-MB, bubbles are synthesized during high stirring and in presence of hydrochloric acid, but due to its water soluble character, no detergent is needed, therefore the crosslinking occurs at the water/air interface (Cavalieri et al., 2006). By slight variations of temperature and pH value in the synthesis phase, the bubbles’ diameter could be changed. PVA-MB were able to produce signal enhancements up to 20 dB in suspensions and have successfully been combined with SPIO nanoparticles to enable multimodal imaging (Grishenkov et al., 2009; Brismar et al., 2012).

Finally, MB can be produced by ink-jet printing using a polyperfluorooctyloxycaronyl-poly(lactic acid) copolymer. The printing method allows to specifically generate bubbles of a defined size and thus studies of swelling or shrinking processes (Böhmer et al., 2006, 2010).

##### Protein-shelled microbubbles

Though less resistant to US waves than polymer-coated MB, but with a longer history of use and development, the first commercially available USCA was the protein-shelled MB Albunex^®^ (Molecular Biosystems, San Diego, CA, USA). On their way to develop a useful USCA for clinical applications, Keller et al. (1987) discovered that a 5% heat-denatured human albumin solution after sonication produces adequately stabilized, air-filled bubbles of mostly less than 10 μm in diameter. First animal experiments showed an enhanced contrast in 2D echocardiography after intravenous injection (Keller et al., 1987) and a behavior similar to erythrocytes to guarantee no interferences in coronary flow or hemodynamics caused by the CA during myocardial US examination (Keller et al., 1988, 1989). Still, Albunex^®^ had a very limited lifetime *in vivo* due to its air-filled core, and the general principle of albumin-shelled MB was soon refined by replacing air with perfluoropropane. This was the beginning of the ‘second generation’ USCA Optison^®^ (GE Healthcare, Buckinghamshire, UK). With a diameter of 2–5 μm, a shell thickness of approximately 15 nm, similar to its predecessor, but consisting of only 1% albumin, clinical trials proved a prolonged and better contrast enhancement compared to Albunex^®^, and a high preference of physicians for Optison^®^ in left ventricular echocardiography (Cohen et al., 1998). Optison^®^ under US application has been used for temporary disruption of the BBB, though side effects like vasoconstriction and hemorrhages might occur with sub-optimal US setting (Hynynen et al., 2001; Raymond et al., 2007). For echocardiography, application of Optison^®^ was found to be generally safe in patients with different cardiologic problems, and potential induction of myocardial necrosis was ruled out (Borges et al., 2002; Wei et al., 2014). Nonetheless, in patients with an unstable cardiopulmonary status or an acute myocardial infarction, application is contraindicated (Dolan et al., 2009). Soltani et al. (2011) tested a mixture of tPA and the CA Optison^®^ and the soft-shell MB SonoVue^®^ (now Lumason^®^, Bracco, Milano, Italy) in a different setup. Using a catheter as a model of human vessels, they treated *in vitro* an acute ischemic stroke via intra-arterial sonothrombolysis, suggesting an effective treatment for some stroke patients (Soltani et al., 2011). Optison^®^ is FDA-approved for cardiac applications such as left ventricular opafication and endocardial border definition. Like polymer-shelled MB, it was shown by Korpanty et al. (2005) to use albumin-based MB, here in combination with dextrose, for molecular targeting. Avidin was incorporated in the shell, so that biotinylated antibodies could be bound functionally to the bubbles (Korpanty et al., 2005). Another more recent approach uses targeted poly-D,L-lactide/albumin hybrid MB for differential diagnosis in patients with chest pain to detect recent ischemia (Leng et al., 2014).

#### Soft-Shell Microbubbles

**FIGURE 1:**
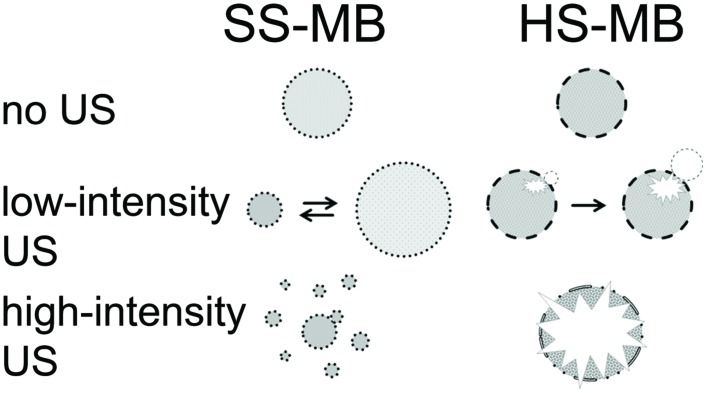
**Behavior of SS-MB (lipid) and HS-MB (polymer) at different US intensities (modified from Hernot and Klibanov, 2008)**.

Soft-shell MB are commonly used for examinations using a low mechanical index (MI) since these MB are sensitively detectable by their non-linear oscillations. The better oscillation properties of SS-MB compared with HS-MB are due to the thinner, more flexible shells, which are held together not by covalent bonding, but hydrophobic interactions. Therefore, after slight shell disruptions, the shell seals itself to minimize surface tension (Borden et al., 2005; Brismar et al., 2012). If sealing is not possible due to the high acoustic pressure, the MB will split into several smaller bubbles instead of bursting like HS-MB (**Figure 1**). To achieve optimal contrast, the shells’ characteristics should be considered and the acoustic power adjusted to the type of MB (Leong-Poi et al., 2002).

The most common shell materials for SS-MB are surfactant molecules or phospholipids, where the length of the acyl chain mainly influences the bubbles’ acoustic dissolution and the monolayers’ cohesiveness (Borden et al., 2005).

##### Phospholipid microbubbles

Several patents from Unger et al. (1994) describe early approaches for the synthesis of SS-MB and handling of gas-filled liposomes with a diameter of approximately 2 μm. Those liposomes were easy to produce by just adding the phospholipid of choice to water or buffer of the temperature slightly above the lipids’ transition point from gel to a liquid crystalline state in which the liposomes form, cooling it back down and removing the liquid by negative pressure application (Unger, 1994). Dried liposomes, in presence of protectants such as trehalose, were found to have a greatly increased shelf life stability and to regain their shape and functionality when refilled with gas again (Crowe et al., 1985). Upscaling production of lipid-layer MB, as well as the use of those bubbles for multimodal imaging via inclusion of paramagnetic particles such as gadolinium in the bubbles for MRI has been patented by D’Arrigo (1993), who also described the potential of MB for tumor treatment, even in the brain (D’Arrigo, 1993).

The first lipid-based USCA that made it to clinical trials and the clinics was Perflutren, sold as Definity^®^ or Luminity^®^ (Lantheus Medical Imaging, North Billerica, MA, USA). It contains perfluoropropane-filled MB in a shell made of three different saturated 16-carbon-long phospholipids. With an average size between only 1–2 μm they are smaller than most HS-MB (Unger et al., 2004). During the trial phase, a good compatibility was seen, as well as left ventricular cavity and myocardial enhancement (Fritz et al., 1997). Despite being developed and FDA approved primarily for echocardiography and cardiologic application, studies showed further clinical applications (Barr, 2013), such as their use to improve detection of tumors, e.g., in the liver. It takes a few minutes to examine a whole liver with US, but the MB lifetime of approximately 3.5 min after bolus injection was found to be sufficient. Compared to non-CEUS, the usage of Definity^®^ showed a higher reliability in tumor- and nodule detection in the liver of rabbits, though the CA itself does not accumulate in the liver (Maruyama et al., 2005).

Similar to HS-MB, lipid-based MB have been found to be useful for therapeutic applications. Integration of lipophilic drugs in the shell, coupling to the outer side of the shell, or encapsulation of therapeutic agents have been shown and are under development for US-assisted and guided therapy, i.e., in cancer treatment. They have also been successfully tested for thrombolysis in combination with US and thrombolytic agents (Unger et al., 2004). More about therapeutical applications will be described later on (see Bubbles as Therapeutics).

Apart from Definity^®^/Luminity^®^, another SS-MB has clinical approval for cardiologic applications. SonoVue^®^ (Bracco Imaging) gained FDA-approval in 2001 and, after a withdrawal, again in 2014, now under the name Lumason^®^. This sulfur hexafluoride filled phospholipid-MB are generally used for left ventricular opafication and endocardial border definition, but in some countries also have approval for general vessel diagnostic or imaging of microvascular structures in the breast or differentiation of lesions in the liver (Claudon et al., 2013; Appis et al., 2015). Apart from that, SonoVue^®^ has also been tested in clinical trials for monitoring of uterine fibroid vascularization and improved ablation (Henri et al., 2014; Jiang et al., 2014).

##### Surfactant-stabilized microbubbles

Hilmann et al. (1985) already suggested the usage of surfactant-stabilized gaseous MB for US diagnostics, but it was not before the mid-90s that this method found its way into more clinically-related *in vitro* research. Among the first surfactant-stabilized MB were those derived from a mixture of the non-ionic surfactants Span60 (sorbitan monostearat) and Tween80 (polyoxyethylene sorbitan monooleate) in different molar ratios, but also other members of the Span/Tween family. MBs of a diameter below the maximum of 10 μm were obtainable and for certain mixtures a shelf time of several weeks and a high echogenicity in B-mode US imaging were shown (Singhal et al., 1993; Wheatley et al., 1994). Still, those MB that succeeded in clinical trials were of different materials. Imagent^®^ (IMCOR Pharmaceuticals Inc., San Diego, CA, USA) MB of approximately 5 μm in diameter, filled with a mixture of air and PFCs, were first tested for renal and liver perfusion studies in rabbits, later for myocardial perfusion and detection of general blood flow abnormalities using Doppler US. It showed promising contrast and compatibility with almost no adverse side effects in first clinical trials (Taylor et al., 1996; Sirlin et al., 1997; Pelura, 1998). Nonetheless other CA from this family dominated and still dominate, both in research and in the clinics. One of them, Levovist^®^ (Schering AG, Berlin, Germany) or SHU-508, was developed in the late 1980s and has been tested and used for several applications since then. Consisting of a saccharide- and palmitic acid-containing shell and air-filled in early versions, it was first tested for examinations of the left ventricle in dogs. A huge advantage was the mean size below 6 μm and the described transpulmonary circulation, making intravenous injections possible and injections directly into the left heart chamber superfluous, (Smith et al., 1989). Clinical trials on echocardiography in patients showed good contrast enhancement and only minor adverse side effects (Schlief et al., 1991). In patients with liver metastases Levovist^®^ greatly enhanced the visualization of blood flow in the tumors, which led to a better differentiation between cancer, hemangiomas and fatty lesions (Ernst et al., 1997). Similarly, more recent studies investigated the advantages of CEUS using Levovist^®^ for differentiation between benign and malignant tumors in several organs, such as spleen, breast, and ovaries. A clear differentiation between malignant and benign tumors was possible, which led to the conclusion that CEUS with Levovist^®^ can help to avoid unnecessary biopsies and surgeries (Ota and Ono, 2004; Wu et al., 2015).

The other main player in the field of surfactant-stabilized MB is Echogen^®^ (Sonus Pharmaceuticals, Bothel, WA, USA) with a core of dodecafluorpentane. First described by Cotter et al. (1994) it quickly became an object of interest (Kronzon et al., 1994). Dodecafluorpentane has a boiling point of approximately 29°C, so it can be injected intravenously as nano-sized, non-echogenic liquid droplets, and immediately transit to echogenic bubbles of 1–2 μm (Forsberg et al., 1994). In direct comparison with Albunex^®^ for echocardiography, the dodecafluorpentane-based CA was found to lead to better results regarding enhancement duration, endocardial border delineation and diagnostic confidence (Grayburn et al., 1998). However, among the surfactant-stabilized MB, Levovist^®^ was the more common, especially since it received approval for clinical applications and in Europe and Canada, whereas Sonus Pharmaceuticals stated withdrawal of their application for FDA approval in 2000 (Correas et al., 2014). By now, Levovist^®^ is not approved anymore for clinical use either. A list of USCA which have/had clinical approval is given in **Table 1**.

**Table 1:** Ultrasound contrast agent that have/had been clinically approved.

Name	First approved for clinical use	Shell material	Gas	Application (examples)	Producer/distributor	Countries
Optison	1998	Cross-linked serum albumin	Octafluoropropane	Left ventricular opafication	GE healthcare, Buckinghamshire, UK	US, Europe
Sonazoid	2007	Phospholipid	Perfluorobutane	Myocardial perfusion, liver imaging	GE healthcare, Buckinghamshire, UK/Daiichi Saniko, Tokyo, JP	Japan, South Korea
Lumason/SonoVue	2001/2014	Phospholipid	Sulphurhexafluoride	Left ventricular opafication, microvascular enhancement (liver and breast lesion detection)	Bracco diagnostics, Milano, Italy	US, Europe, China
Definity/Luminity	2001/2006	Phospholipid	Octafluoropropane	Echocardiography, liver/kidney imaging (Canada)	Lantheus medical Imaging, North Billerica, MA	North America, Europe (approval filed)
Imagent/Imavist	2002, withdrawn	Phospholipid	Perfluorohexane, Nitrogen	Echocardiography, heart perfusion, tumor/blood flow anomalies	Schering AG, Berlin, DE	US
Echovist	1991, withdrawn	Galactose microparticles	Air	Right heart imaging	Schering AG, Berlin, DE	Germany, UK
Levovist	1995, withdrawn	Galactose microparticles, palmitic acid	Air	Whole heart imaging, doppler imaging	Schering AG, Berlin, DE	Canada, Europe, China, Japan
Albunex	1993, withdrawn	Sonicated serum albumin	air	Transpulmonary imaging	Molecular Biosystems Inc., San Diego, CA, USA	Japan, US

### Nanobubbles

Due to their size, MB are unable to leave the vasculature, even in solid tumors, which often have leaky vasculature and a poor lymphatic drainage. This leads to extravasation and retention of macromolecules, also known as the EPR effect (enhanced permeability and retention). To extravasate to the tumor itself, bubbles need to be smaller than 400–800 nm in diameter, therefore referred to as NBs. It has been shown that even bubbles of this dimension were able to produce an enhanced backscatter after US application (Oeffinger and Wheatley, 2004). Additionally, high accumulation of NB in tumors was described, also referred to as passive targeting (Yin et al., 2012). Enhancement of more than 20 dB could be detected for several minutes *in vitro*, using NB of approximately 500 nm in size and being composed of a Span60/Tween80 shell and octafluoropropane as a gaseous core. However, similar to experiments with MB derived from Span60/Tween80, some adverse side effects such as tachycardia were seen in patients due to the limited biocompatibility of the material. Using the highly compatible polyoxyethylene-40-stearate as a substitute for Tween80 to create a “parents suspension” with MB and separation of NB populations by centrifugation led to the development of biocompatible NB of 400-600 nm size. Extravasation into the tumor tissue and contrast enhancement for several minutes was seen using power Doppler (Xing et al., 2010). In different approaches, PLGA and PBCA instead of surfactants have been used to develop biocompatible NB between 150–450 nm (300–500 nm, respectively) in diameter that have been labeled with antibodies binding to HLA-G or TAG-72 to specifically bind to tumors deriving from trophoblastic or epithelial tissue (Xu et al., 2010; Zhang et al., 2014).

Regarding *in vivo* lifetime of NB, results from different studies vary between a few minutes to more than 1 h of contrast enhancement (du Toit et al., 2011; Yin et al., 2012). The size of NB, their material composition and additional coatings or added ligands strongly influence the circulation time and their uptake by the reticuloendothelial system, so that for each NB formulation this factor needs to be determined individually.

If exclusively imaging is wanted, MB still are the first choice due to their higher gaseous content and better oscillation. Nonetheless, accumulation of PFC-containing nanodroplets in the tumor interstitium and US-induced fusion of droplets into MB has been reported (Rapoport et al., 2011). Additionally injection of a nanoparticle emulsion with a generally poor acoustic reflectivity strongly enhances US-contrast when bound in great numbers to specific target sites (Lanza et al., 2000). However, NB are of high interest for therapeutic approaches such as thermal sensitizers in tumors that undergo radiofrequency treatment (Perera et al., 2014). When filled with or coupled to drugs, generally for cancer treatment, the passive targeting to the tumor and the EPR effect might be used for more specific drug delivery and enhanced therapeutic success. Additionally, several groups are also working on the development of targeted, drug-, nucleic acid-filled, or drug-free NBs, directed against different tumor markers.

The current state of the art and preclinical studies with NB will be described later on.

### Silica Shells

With a size between 0.2–2 μm, silica hard-shell particles cannot be placed uniformly in the groups of MB or NB, nor can they be called bubbles, but they are an interesting and new USCA as well. Their rise started around 2009 when Lin et al. (2009) showed their first experiments with gas-filled silica shells *in vitro*. A high MI is required to rupture the shells, releasing the gas of the porous particles and thus produce an US-detectable signal (Lin et al., 2009). Synthesis of silica shells is more complicated compared to other USCA, as it requires templates, generally polystyrene particles, which have to be removed by suitable solvents and replaced by PFCs afterward. After evaluation of possible cytotoxic or hemolytic effects of clinically reasonable concentrations, silica shells were injected in rat testicles and detected with US at varying MI (Hu et al., 2011a). A clearly visible contrast enhancement was seen up to 20 min after injection. In a murine cancer model silica micro- and nano-shells were injected intraperitoneally and imaged using a MI of 1.9 which destroyed the shells and led to detectable “Loss of correlation” signals, when the air sucked out of the particle shell. After image processing steps for motion correction and threshold-, median- and high-pass filter application the high background signals could be subtracted and the accumulation of silica shells in the tumor could be reliably detected (Ta et al., 2012). Liberman et al. (2012, 2014) suggested the usage of silica micro-shells for labeling breast tumors. In their study, the shells were shown to stay in place and being detectable after a local injection for more than 1 day, providing an alternative to guiding wires which are clinical standard. Additionally, those silica particles were found to be promising sonosensitizers in US-mediated ablation therapies in *ex vivo* and some first *in vivo* experiments (Liberman et al., 2012, 2014), whereas another approach combined silica-coated shells with PFC-filling and additionally an included chemotherapeutic drug. With a USCA like that, imaging, ablation and drug delivery would be possible at the same time, but further development is needed here (Ma et al., 2014).

Similar to bubble CA, researchers work on modifications of silica shells for specific targeting. A first attempt of micron-sized shells with a functionalization involved the addition of an amino (-NH_2_) group (Hu et al., 2011b). In their publication they also describe a simple way to control the shells’ diameter and thickness by variation of tetraethylorthosilicate content. Another approach to modify silica particles involves conjugation of hollow silica nano-sized spheres to Gd-DTPA and cycloRGD. This construct has been tested successfully in a murine tumor model for targeted tumor imaging with MRI and US, but animals have only been observed for 8 h after injection and long-term studies will be needed for a proper evaluation (An et al., 2014).

## Biodistribution and Degradation

For the development of new CA, often clinically approved or as biocompatible considered biomaterials are chosen. Parallel to the investigation of the material’s behavior in its bubble/particle shape under US application, *in vitro* toxicity and stability tests under physiological conditions need to be done. Here, first experiments with the CA in presence of blood can be made to rule out potential hemolytic effects of MB under US application (Dalecki, 2005), or a low stability when confronted with blood. For the gaseous filling, it has been shown that PFC as not-naturally in living organisms existing gases, are biologically inert and completely excreted by exhalation within less than 1 day without undergoing modification (Hutter et al., 1999; Hvattum et al., 2001; Toft et al., 2006). Clearance of the shell material happens via the reticuloendothelial system, namely liver and spleen, where macrophages engulf what is left after the gaseous content left the shell by diffusion. Renal clearance has not been reported for clinically approved USCA, which makes CEUS a suitable diagnostic tool also in patients with renal insufficiency, without the need for other examinations and before CA application. Temporary pain in the kidney area after CA injection is most likely caused by the MBs’ accumulation in the small renal vessels, though an effect on blood circulation and function could not be found (Cokkinos et al., 2013; Liu et al., 2013). To the best of our knowledge, a broad comparison study of the exact degradation and clearance processes of the most common USCA has not been done so far, most likely because severe side effects rarely occur and long term impairments have not been described.

Not only degassed, empty shells and fragments, but also intact MB can be taken up by Kupffer cells in the liver, and similar observations have been made for other macrophage populations and neutrophils outside of the liver as well (Kindberg et al., 2003) for Sonazoid^®^ (GE Healthcare, Buckinghamshire, UK). The general tendency to enhance signal in the liver after injection is best known for Levovist^®^, but also Sonazoid^®^ and Optison^®^, and enables detection of liver and spleen anomalies, whereas SonoVue^®^ and Imavist^®^ (Alliance Pharmaceuticals Corp., Chippenham, UK) show little to no uptake by Kupffer cells (Albrecht et al., 2000; Wilson et al., 2000). Due to an accumulation of these CA in the smaller vessels of the liver and a reduced blood flow velocity, both blood pool CA can be used for differentiation of benign and malign liver lesions anyway (Maruyama et al., 2004; von Herbay et al., 2004; Quaia, 2006; Yanagisawa et al., 2007). Shell composition, size and surface properties are responsible for their circulation time and uptake by phagocytes, and no general behavior for phospholipid- or protein-based bubbles can be predicted. So even between materials from the same shell material ‘family’, differences can be seen, most likely due to activation of the complement-system by slightly different surface structures (Chen and Borden, 2011; Kiessling et al., 2012). For unbound bubbles, clearance via the reticuloendothelial system normally happens within the first 10 min after injection (Yuan and Rychak, 2013). To reduce the fast uptake, addition of PEG to the outer surface can help to prolong circulation time and further improve the general biocompatibility (Ryan et al., 2008; Chen and Borden, 2011). For polymer-coated MB, currently not clinically approved, renal excretion after shell breakdown (Palmowski et al., 2008) has been described and for some nanoparticles, excretion by the renal and hepatic system by feces has been shown, though leftovers were still detectable in animals after 3 months (Chen et al., 2013).

Micro- and NB were applied in patients with impaired organ function without mentionable side effects (Albrecht et al., 2004). SonoVue^®^ has been tested in patients with chronic obstructive pulmonary disease without showing more than temporary light impairments like headaches, rushes, or dizziness (Bokor et al., 2001). The theoretical risk of MB injections in patients with coronary diseases is known and premature ventricular contractions have been documented, especially at higher MI and mostly after continuous injection instead of a bolus injection. Adverse effects though are rare and generally mild and temporary, and unlike other imaging modalities, USCA have been safely applied to patients with renal diseases, kidney failure or a generally very bad health condition without an increase in side effects. In at least three cases, however, patients died after SonoVue^®^ injection, but it is uncertain if the CA injections caused the events (van der Wouw et al., 2000; ter Haar, 2009; Wei et al., 2014).

## Active and Passive Targeting

As mentioned before, the EPR effect enables the accumulation of NB in the tissue of tumors, without any specific modifications or targeting molecules. This type of contrast enhancement or drug accumulation in the tissue is therefore referred to as passive targeting (Yin et al., 2012). However, also MB can be used up to a certain point for passive targeting. The chemical composition of the shell affects its behavior in the body, causing accumulations in tissues or attachment to cells. For example, Lindner et al. (2000a) were able to show binding of albumin- and lipid-shelled MB to activated leukocytes adherent to the venular wall without further specific targeting moieties in an inflammation model. Binding was mediated by β2-integrin and the complement system (Lindner et al., 2000a). The same group also demonstrated that incorporation of phosphatidylserine in the lipid shell enables binding to activated leukocytes in inflammation (Lindner et al., 2000b).

**FIGURE 2:**
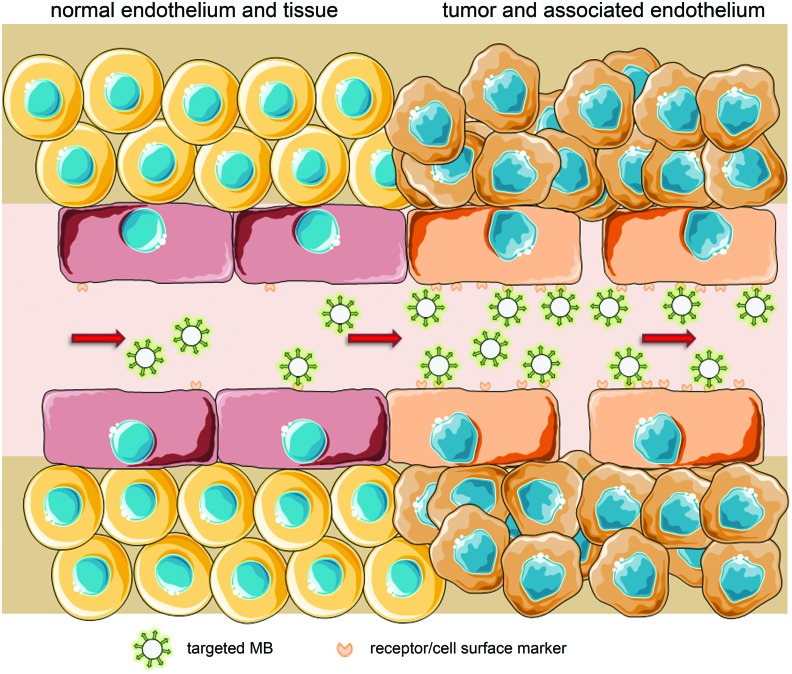
**Active targeting by coupling of ligands to MB that bind to structures overexpressed or exclusively expressed on tumor endothelium (schematic illustration, not drawn to scale)**.

Active targeting requires specific surface modification. Since MB are limited to the vascular compartment, their targets need to be expressed on the luminal side of endothelial cells in pathological environments (**Figure 2**). Therefore, the first approaches toward targeted MB aimed for thrombosis diagnostics and investigated the blood clot dissolving properties of US application. Unger et al. (1998, 2000) used for their first *in vitro* system the shortest functional peptide sequence of fibrinogen, known to bind to glycoprotein IIb/IIIa on platelets, and linked it to a lipid-shell bubble. In a binding assay, they observed both binding and signal enhancement. Their first *in vivo* studies in a dog model showed similarly promising results (Unger et al., 1998, 2000). Other early approaches involved targeting the intercellular adhesion molecule 1 (ICAM-1) for the detection of atherogenesis. Villanueva et al. (1998) used an anti-ICAM-1 antibody covalently bound to a lipid-shell bubble, showing a 40-times higher adhesion of labeled bubbles to interleukin-1β-activated ICAM-1 overexpressing endothelial cells in an *in vitro* flow system, compared to untreated endothelial cells. For a first *in vivo* approach, a rat model of heterotopic heart transplant rejection was chosen. Both successful binding and a strong US contrast enhancement were demonstrated in the transplanted organ undergoing acute rejection (Weller et al., 2003).

A marker of angiogenesis in tumors is integrin α_V_β_3_ expressed by proliferating and activated endothelial cells. To specifically target tumors, antibodies binding to the α_V_-integrin subunit were linked to MB and injected in mice that underwent local growth factor treatment to induce neovascularization at the injection site, and thus endothelial activation. A significantly greater amount of MB was found in this area, as well as a higher acoustic activity (Leong-Poi, 2002). Short peptide sequences like RGD can be used instead of α_V_-integrin-antibodies. In a murine Met-1 breast cancer model, Ellegala et al. (2003) were able to show similar results with MB linked to the integrin-recognition peptide sequence RGD, and suggested α_V_β_3_-targeted MB for early tumor angiogenesis detection. Similar to α_V_β_3_, the vascular endothelial growth factor receptor 2 (VEGFR2) is commonly expressed on activated, proliferating endothelial cells, which makes it another target of interest for the detection of tumor angiogenesis. VEGFR2 was successfully targeted with lipid-shell MB in tumor models (Willmann et al., 2008). However, in a murine model α_V_ and VEGFR2 performed poorly as markers for evaluation of early response to treatment. In this study, endoglin was found to be a more suitable target molecule (Leguerney et al., 2015).

Alternatively, Fokong et al. (2013) linked the peptide sequence IELLQAR to PBCA-MB and achieved a strong contrast enhancement in a murine breast cancer model due to the MB binding to E-selectin, a marker of vascular inflammation and early angiogenesis. Despite many positive pre-clinical studies, evaluation in patients still has to be done. Here, it has to be considered that the recognition motif to the MB which leads to immunogenic responses in patients.

**FIGURE 3:**
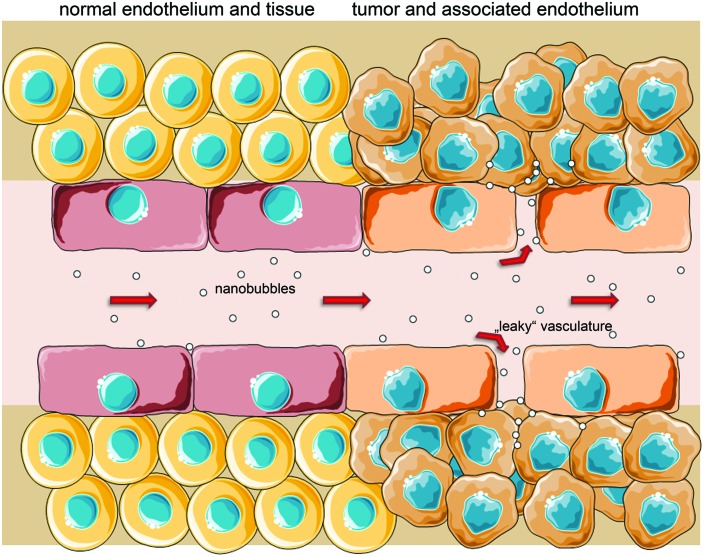
**Passive targeting is enabled by ‘leaky’ vessels with fenestrae up to several 100 nm in tumor-associated endothelium and a poor lymphatic drainage, increasing both likelihood and retention time of nano-sized particles in the interstitium (EPR effect).** After extravasation, NB/particles could also actively target specific surface molecules on cancer cells (schematic illustration, not drawn to scale).

Active targeting has also been tested for NB. The principle is the same, but contrary to MB, NB can also target receptors and other molecules outside the endothelium due to their possible extravasation to the tumor tissue (**Figure 3**). Functional coupling of an antibody, in this case directed against the TAG-72 antigen overexpressed on several epithelial tumors, to polymer-coated NB and successful binding of those to cancer cells *in vitro* has been shown by Xu et al. (2010). The usefulness of such an approach for tumor treatment has also been demonstrated *in vivo* using HLA-G as a target for antibody-coupled polymer-NB (Zhang et al., 2014). The combination of HIFU and the local release of chemotherapeutics after bubble disruption was shown to strongly enhance apoptosis of tumor cells. Recently Jiang et al. (2015) presented a promising new construct for tumor imaging, improving the idea and the *in vitro* model of Liu et al. (2007). Their lipid-based NB were coupled to Herceptin^®^ (Roche, Basel, CH), also known as trastuzumab, a therapeutic monoclonal antibody binding to Her-2, which is overexpressed in many breast tumors. They found a significant increase in NB-binding to the tumor in xenograft-models which are known to overexpress Her-2, but only little accumulation in tumor xenografts with low Her-2 expression. Targeted NB in Her-2 overexpressing tumors also had an increased washout half-time, enabling longer observation times. Additionally, their bubble-construct was found to be of a low cytotoxicity (Jiang et al., 2015). To this date, this might be the approach being the closest to clinical studies. Herceptin^®^ has also been conjugated to mesoporous silica nanoparticles and showed tumor-specific toxicity *in vitro*, suggesting further development of silica particles in the field of targeted imaging and drug delivery (Milgroom et al., 2014).

## Bubbles as Therapeutics

The basic idea behind using MB for therapeutic purposes is their ability to enhance vibration-effects generated by US pulses. Already Tachibana and Tachibana (1995) observed that US-mediated thrombolysis is more effective in the presence of bubbles, resulting from acoustic cavitation of the sonicated bubbles. Then, Molina et al. (2006) could demonstrate in a clinical study on stroke patients that the rate of complete arterial recanalization after sonification was significantly higher in the group that received MB and tPA, compared to only tPA. Similar results were found in a sonothrombolysis trial with transcranial US (Molina et al., 2009). Thus, the application of MB to enhance US-mediated thrombolysis has the potential for improving therapy of acute stroke patients (Tsivgoulis et al., 2010). Recent results from Petit et al. (2015). support the claim of strong synergistic effects when US, MB, and tPA are combined due to an enhanced clot lysis and degradation of fibrin.

Another application area for USCA as therapeutics is HIFU-based tumor ablation. In the non-contrast-enhanced setting, this is facilitated by thermal and mechanical effects in the target tissue arising from absorption of high-frequency US waves and the internal conversion into heat and vibration, including acoustic cavitation and radiation forces (Saha et al., 2014). The group of Jiang et al. (2014) could demonstrate in a clinical study that the additional administration of MB (SonoVue^®^) to patients prior to HIFU ablation increased the HIFU-mediated tumor ablation significantly more than solely HIFU, even at lower sonification power and less sonification time. The mechanism of the bubble-enhanced HIFU-mediated tissue ablation is suspected to be a result of the violent bubble collapse, which may cause mechanical injury in the target tissue (Farny et al., 2010).

A further application area for USCA as therapeutics is sonoporation. LOFU pulses mediate temporal permeabilization of cell membranes and enable drug delivery across biological barriers like blood vessels or even the BBB (Kiessling et al., 2012). Without bubbles, the low-intensity acoustic US-field promotes inertial cavitation, resulting in gas bubble creation, leading to streaming and radiation forces to the tissue. The addition of MB decreases the energy required to create cavitation and increases the effectiveness of cell membrane permeation and of cell transfections (Delalande et al., 2013).

Microbubbles might also be used for gene therapy. Here, the transport of genes or nucleic acids to and their introduction into cells is facilitated by CEUS. Wang et al. (2008) showed that in presence of MB the efficacy of cell transfection was 1–2 orders of magnitude higher than with plasmid DNA alone. However, the efficacy of US-mediated gene transfer is low in comparison to electroporation or viral transfection. Thus, the group around Sun intends to design MB, which are better suited for gene delivery than the commercially available Definity^®^. They increased the lipid-shell acyl-chain length to enhance the bubble stability or charged the spheres’ shell positively in order to improve DNA-binding affinity. Using the designed MB for gene transfection, the group observed significantly enhanced transfection effectiveness by stronger transgene expression compared to gene transfection with Definity^®^. Still, transfection efficacy is not comparable to viral transfection, but since US-mediated transfection using MB contains less risks regarding immunotoxicity and possible oncogenic effects and provides a better spatial and temporal control of the process, work in this field will be continued (Sun et al., 2014). Dewitte et al. (2014) also used a combination of lipid MB and nucleic acids for transfections. In their setup, mRNA coding for cancer antigens was bound to the lipid bubbles. Application of US led to transfection of murine dendritic cells *ex vivo*. These were later on injected, similar to a vaccination, into lymph nodes near the tumors of nude mice. The maturated antigen-presenting dendritic cells then lead to a strong reduction of tumor mass, in some cases even complete remission and long-term immunity to tumors expressing this antigen (Dewitte et al., 2014). Similar to transfection studies with MB, Yin et al. (2014) used NB to transfect cells *in vitro* and *in vivo* with siRNA to enhance apoptosis in tumors after NB injection and US treatment with promising first results.

**FIGURE 4:**
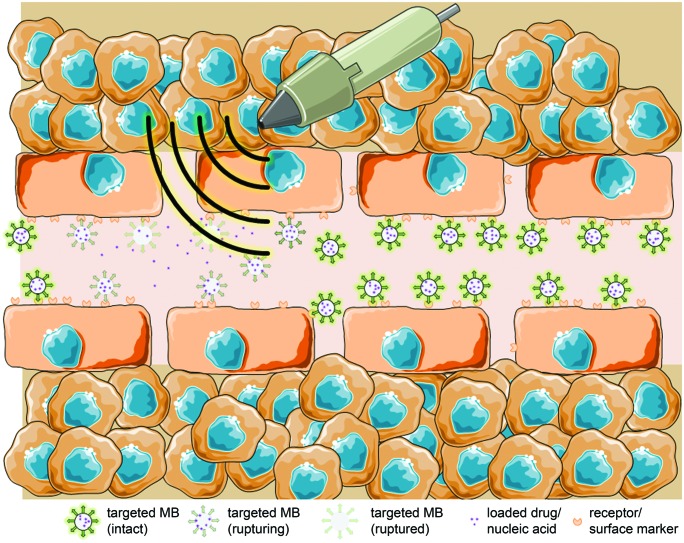
**Targeted MBs with entrapped drug/nucleic acids rupture under the US-induced acoustic pressure and release their loading specifically at the side of a tumor (schematic illustration, not drawn to scale)**.

**FIGURE 5:**
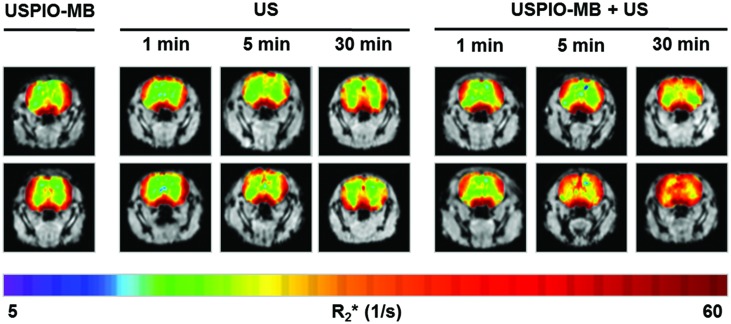
**Effect of US only (US) and USPIO-labeled MB with US (USPIO-MB+US) on the BBB.** T_2_^∗^-weighted MRI images were taken before and after US/USPIO-MB+US application, R_2_^∗^ values of each measurement were color-coded and overlayed. The most striking difference can be seen after US application for 30 min, comparing pre- and post-scan in both groups (from: Lammers et al., 2015, ©2014 WILEY-VCH Verlag GmbH & Co. KGaA, Weinheim).

The second application field for bubble-enhanced sonoporation is US-mediated drug release (**Figure 4**). Using drug-loaded MB, it is possible to spatiotemporally apply LOFU pulses at certain organs to increase the membrane permeability, and at the same time increase the energy to promote bubble collapse and particle release (Tzu-Yin et al., 2013). This way, the group around Kiessling and co-workers used USPIO containing PBCA-MB (USPIO-MB) as a proof of principle approach to demonstrate drug delivery across the BBB in mice (**Figure 5**). For the experiment, mice were scanned by MRI before and after injection of USPIO-MB and successive US treatment in the head region using destructive pulses. In comparison to control groups, the USPIO brain incorporation was significantly increased in the group receiving US treatment. Drug delivery across the BBB was additionally confirmed by histology, and significantly enhanced FITC-dextran extravasation and deposition within the brain was detected in animals receiving USPIO-MB and sonification. The temporal and spatial BBB-opening offers a new perspective to treat gliomas, but also neurodegenerative disorders like Alzheimer or Parkinson’s disease, where macromolecular drugs or growth factors have to be locally accumulated (Lammers et al., 2015). However, also outside of the brain US-supported drug delivery is promising. In an animal model of incomplete tumor resection, cetuximab-loaded MB and US stimulation had stronger therapeutic effects on the remaining tumor mass than cetuximab-only treated animals, which was confirmed by *in vivo* fluorescence and bioluminescence, as well as caliper measurements (Sorace et al., 2014). Co-delivery of paclitaxel and siRNA to inhibit anti-apoptotic protein production also showed very promising results in a murine HepG2 tumor model. Here, tumor growth was strongly inhibited in animals which received NB with paclitaxel entrapped in and siRNA-containing micelles attached to the shell, additionally to US application. Furthermore those animals showed longer survival (Yin et al., 2014). Despite those promising results further animal studies need to be done before similar setups can go into clinical trials.

Besides being or delivering the therapeutic agents themselves, MB were shown to be highly useful in monitoring of oncological therapies. Shortly after chemotherapy started, Lassau et al. (2005, 2006). could show changes in tumor vascularity or increasing necrosis in patients with metastatic melanoma and gastrointestinal stroma tumors after injection of Levovist^®^ and SonoVue^®^. Using standardized dynamic CEUS measurements, the group was even able to predict the outcome of antiangiogenic tumor therapy by evaluating the “area under the perfusion curve.” Among several criteria, this one was found to be highly correlated to therapy response and freedom from progression (Lassau et al., 2014). Differentiation of responders and non-responders at early points in time is needed to plan if the therapy will be continued or if an alternative has to be considered.

## Conclusion

In this review we presented the historical development of CAs for US imaging from the early steps to the current state of the art. MBs are established CA for clinical vascular analysis and liver diagnosis, but also other applications. A broad variety of materials and sizes generates the basis for those multiple diagnostic and therapeutic applications. Currently, there are three clinically approved CAs, but with many on-going pre-clinical studies and promising first results, more clinical trials can be expected to start within the next years. The combination of US CAs for diagnostics and therapy in one single injection therefore holds a great potential for the future and might be a valuable tool for treatment of widespread and deadly diseases like cancer or cardiovascular diseases. But also for neurodegenerative diseases USCA might play a growing role in treatment. A temporary increase in vessel permeability could enhance drug delivery to the brain or other tumors while reducing systemic side effects due to the mostly local delivery. USCA might also gain more importance in non-invasive ablation therapies.

When functional analysis or tissue vascularization does not suffice, the application of molecularly-targeted CA would be complementary, both in diagnosis and targeted drug delivery. For targeted MB and NB, the pre-clinically common biotin-streptavidin linking method needs a similarly strong and easy alternative to avoid immune reactions. Additionally, more targets with a sufficiently strong binding to the ligand are required, otherwise the shear stress in vessels might inhibit successful binding of the CAs and thus impair the contrast enhancement. Another point that might need refinement is the CA’s circulation time. So far even PEGylated bubbles with PFC have a very limited lifetime, limiting the time frame for examination. But with no described long term impairments after injection, rare side effects and a valuable role in diagnostics, sonothrombolysis, monitoring of treatment, specific and targeted substance delivery vesicles or sonosensitizers, the technology of CA and US in combination is –with some specific adjustments- the swiss army knife among CAs and imaging modalities.

## Conflict of Interest Statement

The reviewer Claus Christian Glüer declares that, despite having collaborated with the author Fabian Kiessling, the review process was handled objectively. The authors declare that the research was conducted in the absence of any commercial or financial relationships that could be construed as a potential conflict of interest.

## Abbreviations

BBB: blood-brain barrier
CA: contrast agent(s)
CEUS: contrast-enhanced ultrasound
HIFU: high-intensity focused ultrasound
HS-MB: hard-shell microbubble(s)
LOFU: low-intensity focused ultrasound
MB: microbubble(s)
NB: nanobubble(s)
PBCA: poly(n-butylcyanoacrylate)
PEG: polyethylene glycol
PFC: perfluorocarbon(s)
PLGA: poly(D,L-lactide-co-glycolide)
PVA: poly(vinyl-alcohol)
SS-MB: soft-shell microbubble(s)
tPA: tissue plasminogen activator
US: ultrasound
USCA: ultrasound contrast agent(s)
(U)SPIO: (ultrasmall) superparamagnetic iron oxide.
